# Paroxysmal and unusual symptoms as first clinical manifestation of multiple sclerosis do not indicate benign prognosis—The PaSiMS II study

**DOI:** 10.1371/journal.pone.0181458

**Published:** 2017-07-27

**Authors:** Gabriel Bsteh, Rainer Ehling, Lisa-Maria Walchhofer, Harald Hegen, Michael Auer, Sebastian Wurth, Franziska Di Pauli, Michaela Wagner, Markus Reindl, Florian Deisenhammer, Thomas Berger

**Affiliations:** 1 Department of Neurology, Medical University of Innsbruck, Innsbruck, Austria; 2 Department of Neurology, Clinic for Rehabilitation Muenster, Muenster, Austria; 3 Department of Neuroradiology, Medical University of Innsbruck, Innsbruck, Austria; University of Oxford, UNITED KINGDOM

## Abstract

**Background:**

Paroxysmal (PS) and unusual symptoms (US) account for approximately 1.6% of initial manifestations of multiple sclerosis (MS) and have comparable conversion rates to clinically definite MS (CDMS) as classical bout onset symptoms (CS). However, long-term prognosis and clinical outcome of patients experiencing PS or US as first clinical manifestation are unclear.

**Methods:**

Clinical, MRI and cerebrospinal fluid data were obtained retrospectively and patients presenting with PS or US were compared to patients with CS presentation.

**Results:**

In a cohort of 532 relapsing onset MS patients followed for a mean period of 11.4 years (SD 3.6), 10 (1.9%) patients initially presented with PS/US. PS/US patients received disease modifying treatment (DMT) in a significantly smaller proportion immediately after the first clinical symptom (30% vs. 61.7%; p = 0.021) and during the observation period (60% vs. 83.5%; p = 0.033). In multivariate models correcting for sex, age at initial symptoms, complete remission of initial symptoms, total number of T2 and contrast-enhancing lesions, presence of oligoclonal bands and DMT exposure, PS/US were not associated with lower annualized relapse rate or lower EDSS over time.

**Conclusion:**

In addition to a similar conversion rate to CDMS, patients presenting with PS/US at disease onset display very similar relapse and disability rates as patients with CS onset. Consequently, initial presentation with PS/US does not indicate benign or atypical MS, but requires DMT initiation based on the same criteria as in CS patients.

## 1. Introduction

Paroxysmal symptoms (PS) are brief (lasting seconds to minutes) symptoms occurring suddenly and many times a day. They are often stereotyped and continue in clusters with great intensity for days up to a few months[1]. While their pathophysiology is well characterised, they are among the most frequently misinterpreted manifestations of multiple sclerosis (MS) (1). Since the latest revision of the definition of a MS relapse, PS (historical or current) are now accepted as relapses as long as they consist of multiple episodes occurring over not less than 24 hours [2]. “Unusual symptoms” (US) are thought to represent the clinical correlate of mainly cortical MS lesions [3,4]. Most frequently, focal epileptic seizures and various types of aphasia are classified as US in MS [1]. In a previous large scale study, **P**aroxysmal **a**nd Unusual **S**ymptoms **i**n **MS** (PaSiMS I), we found a prevalence rate of 1.1% and 0.5% for PS and US as initial manifestation of MS, respectively [5]. Prediction of long-term prognosis at disease onset is generally challenging in MS. Female sex, young age, complete remission of initial symptoms, absence of oligoclonal bands (OCB), low T2 lesion load and absence of contrast-enhancing (CE) lesions have consistently been identified as predictive factors for favourable long-term outcome [6–12]. Recently, we have demonstrated that PS and US are associated with the same risk of developing CDMS as patients with classical bout onset, but data regarding long-term prognosis in this particular subgroup of MS patients is still lacking [5]. There is evidence regarding effectiveness of various disease modifying treatments (DMT) such as interferon ß, glatiramer acetate, natalizumab, fingolimod, teriflunomide, dimethylfumarate, alemtuzumab, daclizumab and ocrelizumab in delaying the diagnosis of CDMS and/or reducing the annualized relapse rate (ARR) and disability progression [13–22]. However, patients presenting with PS or US have either not been included [13,15] or not reported [14,16–22] in these studies.

In this light, data on prognosis and outcome of patients presenting with a PS or US at MS disease onset are needed. The objective of the present study (PaSiMS II) was therefore to investigate the long-term prognosis and outcome of patients experiencing PS or US as first clinical manifestation of MS.

## 2. Material and methods

### 2.1 Protocol, confidentiality and participants

As described previously, an electronic database was established at the MS Clinic of the Department of Neurology, Medical University of Innsbruck, which is the reference centre for MS in Western Austria. Data were collected retrospectively from patient’s charts until 1987 and prospectively from 2004 onwards [11].

Confidentiality and data protection are ensured in keeping with the recommendations of the declaration of Helsinki and the Austrian Data Safety Authority instructions (www.ris.bka.gv.at., 2016). The study was approved by the ethics committee of the Medical University Innsbruck. Informed written consent was obtained from every patient. By December 2016, a cohort of 1,859 MS patients has been included according to McDonald diagnostic criteria [2,23]. The prevalence of MS in Austria is 148 per 100.000 people [24]. Given a population of about 1.6 million people in Western Austria, this database is likely to include most MS patients from this geographic area [25].

**Fig 1:**
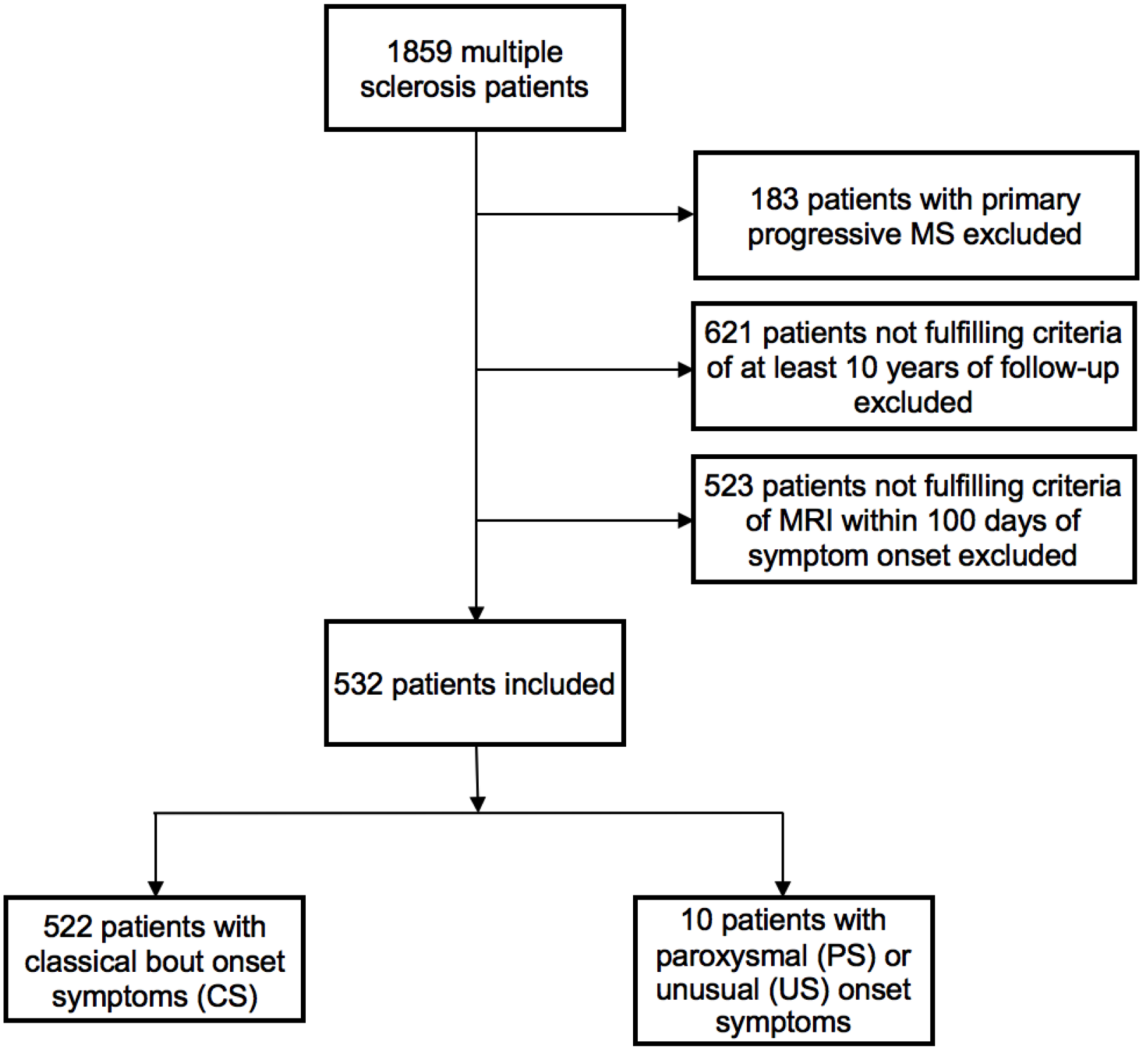
Study flow chart. MS: multiple sclerosis. MRI: magnetic resonance imaging.

For PaSiMS II, patients with a clinically isolated syndrome (CIS), relapsing-remitting (RR) or secondary progressive (SP) MS and a minimum follow-up period of ten years were included. Since the definition of PS excludes a continuous neurological progression, patients exhibiting a primary progressive disease course were excluded. In order to be able to account for the influence of the number of T2 and CE lesions, we also excluded all patients who had not received cerebral MRI within 100 days of reported symptom onset (Fig 1).

Similar to PaSiMS I, patients were divided into two subgroups according to the initial clinical presentation: 1) PS (occurring frequently over at least 24 hours) or US (focal epileptic seizures) and 2) classical bout onset symptoms (CS) [5].

Brain MRI scans were done on 1.5 T or 3 T MR scanners. MRI protocols differed in detail but included axial and sagittal T2 sequences and coronal MPRage before and after Gadolinium. MRI scans were rated according to Barkhof-Tintoré criteria [26,27] by two experienced neuroradiologists (LMW and MW), who were blinded for clinical data. Furthermore, status of OCB was included.

### 2.2 Statistical analysis

Variables are presented as absolute number and percentage, mean and standard deviation, median and inter-quartile range (IQR) or median and minimum-maximum range as appropriate. Differences between patient groups in categorical variables were evaluated using chi-square test. Numeric variables were analysed by independent t-test or Mann-Whitney-U test depending on normal distribution tested by Kolmogorow-Smirnow-test. Log-rank test was used to compare time to first relapse in the investigated subgroups. The influence of PS/US on ARR and disability (EDSS) was evaluated by binary logistic regression models correcting for sex, age at initial symptoms, remission of initial symptoms, total number of T2 and CE lesions, OCB positivity and exposure to DMT. As cut-off values for binary logistic regression, we used the upper tertile for ARR (≥/< 0.4 during the observation period) and EDSS ≥/< 4 (reflecting occurrence of gait impairment as the first significant milestone of MS associated disability) after 10 years [28,29]. Significance was based on a p-value of <0.05. Data were analysed using SPSS 23.0 (SPSS Inc, Chicago, IL, USA).

## 3. Results

A total of 532 RRMS and SPMS patients have been included (Fig 1). Ten patients (1.9%) presented with PS or US. PS presentations included trigeminal neuralgia (n = 1), occipital neuralgia (n = 1), paroxysmal diplopia (n = 2), dysarthria and/or hemiataxia (n = 1) and tonic spasms (n = 1). US presentations included focal epileptic seizures with secondary generalization (n = 2), de-novo status epilepticus (n = 1) and repetitive transient speech arrest (n = 1).

Clinical, MRI and cerebrospinal fluid (CSF) characteristics of patients presenting with PS/US compared to patients presenting with CS are given in Table 1. There were no significant group differences regarding demographic characteristics, OCB status or disease course.

**Table 1:** Clinical, MRI and CSF characteristics of patients presenting with PS/US compared to CS.

	*PS/US onset*	*CS onset*	*p*
*N*	10	522	
*Clinical data*			
Female^1^	8 (80.0)	386 (73.9)	0.255^a^
Age at first symptom (years)^2^	32.1 (9.9)	29.5 (8.9)	0.369^b^
Follow-up (years)^2^	11.2 (5.3)	11.4 (4.4)	0.299^c^
Complete remission after 6 months^1^	9 (90)	410 (78.5)	0.337^a^
Brain MRI*			
Time to MRI (days)^2^	37 (33.8)	34 (29.7)	0.232^c^
Number of T2 lesions^3^	9 (6–34)	13 (6–23)	0.128^c^
Number of CE lesions^3^ **	0 (0–1)	0 (0–1)	0.968^c^
Barkhof-Tintoré criteria fulfilled	8 (80)	409 (78.2)	0.418^a^
*CSF analysis*			
positive OCB	10 (100%)	469 (93.2)***	0.510^a^
*Diagnosis at last follow-up*			0.209^a^
CIS	1 (10)	22 (4.2)	
RRMS	8 (80)	461 (88.3)	
SPMS	1 (10)	39 (7.5)	
*DMT*			
DMT exposure prior to first relapse^1^	3 (30)	322 (61.7)	0.021^a^
DMT exposure during observation period^1^	6 (60)	436 (83.5)	0.033^a^
Time to DMT^2^ (years)	2.9 (1.9)	2.1 (1.5)	0.172^c^
Time on DMT^2^ (years)	4.8 (4.0)	5.8 (6.9)	0.486^c^
*Disease course*			
Time to diagnosis (McDonald 2010) (years)^2^	2.2 (1.9)	1.9 (2.2)	0.459^b^
Time to first relapse (CDMS)	2.2 (2.4)	2.1 (2.3)	0.972^d^
ARR during observation period^2^	0.19 (0.21)	0.48 (0.31)	0.003^c^
EDSS 10 years after onset^4^	1.5 (0–6)	2.5 (0–8)	0.105^c^

^1^absolute number and percentage;

^2^mean and standard deviation;

^3^median and inter-quartile range;

^4^median and minimum-maximum range

*performed within 100 days after symptom onset.

**available from 473 patients.

*** available from 503 patients.

Analysed with:

^a^ chi-square test,

^b^ independent t-test.

^c^ Mann-Whitney U test;

^d^ Log-rank test

MRI: magnetic resonance imaging; CSF: cerebrospinal fluid; PS: paroxysmal symptom; US: unusual symptom; CS: classical bout onset; CIS: clinically isolated syndrome; RRMS: relapsing-remitting multiple sclerosis; SPMS: secondary progressive multiple sclerosis; CE: contrast-enhancing; OCB: oligoclonal bands; DMT: disease modifying treatment; CDMS: clinically definite MS; ARR: annualized relapse rate; EDSS: expanded disability status scale.

Although the median total number of T2 lesions on initial MRI was lower in the PS/US group (9; interquartile range (IQR) 6–34) compared to the CS group (13; IQR 6–23), this difference did not reach statistical significance (p = 0.128). In univariate analysis, PS/US patients did receive DMT in a significantly smaller proportion immediately after the first clinical symptom (30% vs. 61.7%; p = 0.021) and during the observation period (60% vs. 83.5%; p = 0.033). Mean time to McDonald MS and also to first relapse was similar in both groups. In addition, when split into the groups: PS, US and CS there was also no significant diagnostic delay in time to diagnosis of MS (2). (S1 Table) However, the annualized relapse rate (ARR) during the observation period was significantly lower in the PS/US group (0.19 vs 0.48; p = 0.003). Median EDSS 10 years after initial manifestation also was lower in PS/US patients (1.5 vs 2.5), but did not reach statistical significance (p = 0.105).

### Multivariate analysis of ARR and EDSS

In a multivariate model, higher age at initial symptoms, lower number of T2 lesions, lower number of CE lesions, OCB negativity and exposure to DMT were independent predictors of an ARR <0.4 during the observation period, while PS/US at disease onset was not. (Table 2)

**Table 2:** Multivariate models of annualized relapse rate over the observation period and EDSS 10 years after symptom onset.

	Annualized relapse rate ≥ 0.4			EDSS ≥ 4 ten years after symptom onset		
	OR^1^	95% confidence interval	P value	OR^1^	95% confidence interval	P value
PS/US onset	0.78	0.45–1.05	0.123	0.91	0.23–1.71	0.769
Female	1.38	0.91–2.11	0.133	0.93	0.87–0.99	0.049
Complete remission after 6 months	1.10	0.68–1,79	0.691	0.85	0.73–0.96	0.023
Age at initial symptoms (per 5 years)	0.96	0.94–0.98	0.038	1.09	1.05–1.13	0.041
Number of T2 lesions (per lesion)	1.01	1.00–1.02	0.031	1.12	1.09–1.15	0.032
Number of CE lesions (per lesion) *	1.32	1.21–1.43	0.003	1.31	1.14–1.57	0.018
Positive OCB **	0.77	0.36–1.67	0.513	1.29	0.86–1.77	0.436
DMT exposure	0.49	0.34–0.73	<0.001	0.90	0.81–0.98	0.025

^1^Calculated by binary logistic regression models: OR values between > 1 indicating higher risk of an annualized relapse rate ≥ 0.4 over the observation period and an EDSS ≥ 4 10 years after symptom onset, respectively.

* available from 473 patients;

** available from 503 patients.

EDSS: expanded disability status scale; PS: paroxysmal symptom; US: unusual symptom; CE: contrast-enhancing; OCB: oligoclonal bands; DMT: disease modifying treatment; OR: odds ratio.

Younger age at first symptom, complete remission of initial symptoms after 6 months, lower number of T2 lesions, lower number of CE lesions, OCB negativity and exposure to DMT were independently associated with a lower risk of reaching an EDSS ≥4 ten years after initial symptoms. However, initial clinical presentation as a PS/US was not found to be prognostic for later EDSS.

## 4. Discussion

In this retrospective study on a large cohort of relapsing onset MS patients, we aimed to investigate long-term prognosis and outcome of patients experiencing PS or US as first clinical manifestation.

We found that—after correcting for known, commonly agreed prognostic factors in MS—PS/US patients displayed very similar annualized relapse rates and disability (EDSS) compared to patients presenting with CS after an observation period of more than ten years.

In the previous PASiMS I study, which was designed to establish the prevalence and clinical features of PS and US, PS patients exhibited fewer relapses and less EDSS progression than CS patients. We had already hypothesised that these differences were possibly due to differences in baseline T2 lesion load, but in the absence of MRI data for CS patients we were previously not able to correct for these potential confounders [12].

In the present PASiMS II study, which now included MRI data from CS patients, we were able to replicate these findings. However, after correcting for the number of T2 and CE lesions, DMT exposure and known prognostic factors for MS disease course, such as sex, age, complete remission of initial symptoms and OCB status, PS/US were not found to have an additional effect on relapse rate and degree of disability after 10 years. Therefore, PS/US *per se* seems not to indicate a favourable prognosis.

Similar to the PASiMS I study, a substantial part of patients with non-classical bout onset symptoms presented with epileptic seizures. This observation is potentially attributable to a higher risk for epileptic seizures in MS patients that has been reported in several epidemiological studies previously [30,31]. However, patients presenting with epileptic seizures as first clinical symptoms of MS did not differ in the time to MS diagnosis from patients presenting with PS. (S1 Table) In addition, both PS and US as first clinical symptom of MS were found to be associated with a less frequent exposure to DMT than CS prior to a first relapse, but also during the total observation period.

This trend, albeit not statistically significant, was already evident in PaSiMS I. In contrast to the present study, where the availability of brain MRI within 100 days after initial symptom onset was a key inclusion criterion, patients were included in PaSiMS I irrespective of their MRI status, resulting in a larger sample size. As a consequence, a significant subgroup of patients was not diagnosed with MS before the first relapse and had therefore not even the chance to receive DMT before conversion to CDMS, leading to a lower DMT initiation rate after the first clinical symptom in the total cohort. We were able to corroborate this bias by the finding of a significantly higher DMT exposure rate prior to first relapse in the finally included PaSiMS II cohort as compared to patients who had to be excluded because they had not received MRI within 100 days of initial symptom onset (S2 Table).

We found a very similar distribution of PS/US and CS patients with respect to different eras of DMT usage making a potential bias due to changing DMT availability and prescription rates highly unlikely (S3 Table).

Therefore, DMT seems to be initiated more hesitantly in PS/US patients compared to CS patients. The most likely reason for this hesitancy is probably the transient nature of PS/US itself, whereas typical MS onset symptoms may be more disabling and, thus, might urge earlier DMT initiation than PS/US. However, as evidenced in our study, the long-term clinical disease course of patients experiencing PS/US as first clinical episode of MS is not different from patients having CS. The efficacy of DMT in preventing or delaying time to CDMS and/or reducing the ARR and disability progression in classical bout onset MS has been well established [13–22]. The benefit of early treatment has been shown to also sustain in long-term follow-up studies [32]. Therefore, patients initially presenting with PS/US should be thoroughly assessed and counselled regarding DMT initiation based on clinical, MRI and CSF findings identically to CS patients.

Our study has limitations due to its retrospective design, possibly introducing a potential bias. However, data collection was primarily conducted prospectively [5,11]. The inclusion criteria of a minimum documented follow-up of 10 years may have led to an underestimation of disability, since patients with severe disease course tend to be lost to follow-up. However, this potential effect is attributable for both the PS/US and CS group in our cohort deeming a substantial influence on our results unlikely. Another limitation is the small number of patients in the PS/US group which is due to the rarity of PS/US as initial manifestation of MS and reduces the statistical power of our models.

In conclusion, initial presentation with PS/US does not indicate a favourable or even benign MS prognosis. Hence, identifying PS and US as possible first clinical symptoms of MS is paramount. DMT should be considered early and treatment decisions should be based on the same criteria as in CS patients.

## Supporting information

S1 Table Time to MS diagnosis. (DOCX)

S2 Table DMT exposure prior to first relapse depending on MRI within 100 days after initial symptoms. (DOCX)

S3 Table Timeframes of onset of initial symptoms. (DOCX)

S4 Table Study’s underlying data set (PaSiMS II cohort). (XLSX)
