# Supplementary material for: Paroxysmal and unusual symptoms as first clinical manifestation of multiple sclerosis do not indicate benign prognosis—The PaSiMS II study
Source: PLoS One. 2017 Jul 27;12(7):e0181458. doi: 10.1371/journal.pone.0181458 (PMC5547697; doi:10.1371/journal.pone.0181458)
Supplement: S1 Table — (DOCX) [file pone.0181458.s001.docx]

**S1 Table. Time to MS diagnosis.**

|  | PS onset | US onset | CS onset | *p*^a^ |
| --- | --- | --- | --- | --- |
| Time to diagnosis^2^ (McDonald 2010) (years) | 2.1 (1.3) | 2.3 (2.6) | 1.9 (2.2) | 0.674 |

^1^mean and standard deviation

Analysed with ^a^ independent t-test

PS: paroxysmal symptom; US: unusual symptom; CS: classical bout onset.
