# Supplementary material for: Paroxysmal and unusual symptoms as first clinical manifestation of multiple sclerosis do not indicate benign prognosis—The PaSiMS II study
Source: PLoS One. 2017 Jul 27;12(7):e0181458. doi: 10.1371/journal.pone.0181458 (PMC5547697; doi:10.1371/journal.pone.0181458)
Supplement: S2 Table — (DOCX) [file pone.0181458.s002.docx]

**Supplemental Table 2. Timeframes of onset of initial symptoms.**

|  | PS/US | CS | p-value |
| --- | --- | --- | --- |
| Date of initial symptoms before 1997 (before treatment era) | 1 (10%) | 40 (7.6%) | 0.673 |
| Date of initial symptoms 1997-2003 | 3 (30%) | 187 (35.8%) | 0.734 |
| Date of initial symptoms 2004 and later | 6 (60%) | 295 (56.6%) | 0.873 |
